# Autocitrullination confers monocyte chemotactic properties to peptidylarginine deiminase 4

**DOI:** 10.1038/s41598-023-34469-1

**Published:** 2023-05-09

**Authors:** Ken Yoshida, Haruyasu Ito, Daisaburo Kurosaka, Ryo Ikeda, Kentaro Noda, Mitsuru Saito, Daitaro Kurosaka

**Affiliations:** 1 Division of Rheumatology, Department of Internal Medicine, The Jikei University School of Medicine, 3-25-8 Nishi-Shimbashi, Minato-ku, Tokyo, 105-8461 Japan; 2 Department of Orthopaedic Surgery, The Jikei University School of Medicine, 3-25-8 Nishi-Shimbashi, Minato-ku, Tokyo, 105-8461 Japan

**Keywords:** Immunology, Rheumatology

## Abstract

Peptidylarginine deiminase 4 (PAD4) contributes to the production of citrullinated proteins as autoantigens for anti-citrullinated protein antibodies (ACPAs) in rheumatoid arthritis (RA). PAD4 can also self-deiminate via autocitrullination. However, the role of this process in RA pathogenesis has not been elucidated. This study aimed to clarify PAD4 function before and after autocitrullination and identify citrullinated PAD4 in the synovial fluid of patients with RA. The autocitrullination of recombinant human PAD4 (rhPAD4) was catalyzed in vitro and determined using anti-modified citrulline immunoblotting. Monocyte chemotaxis was evaluated using Boyden chambers, and citrullinated rhPAD4’s ability to induce arthritis was assessed in a C57BL/6J mouse model. Citrullinated PAD4 levels were measured in the synovial fluid of patients with RA and osteoarthritis using a novel enzyme-linked immunosorbent assay. Chemotactic findings showed that citrullinated rhPAD4 recruited monocytes in vitro, whereas unmodified rhPAD4 did not. Compared to unmodified rhPAD4, citrullinated rhPAD4 induced greater inflammation in mouse joints through monocyte migration. More citrullinated PAD4 was found in the synovial fluid of patients with RA than in those with osteoarthritis. Citrullinated PAD4 was even detected in ACPA-negative patients with RA. The autocitrullination of PAD4 amplified inflammatory arthritis through monocyte recruitment, suggesting an ACPA-independent role of PAD4 in RA pathogenesis.

## Introduction

Rheumatoid arthritis (RA) is a systemic and chronic inflammatory disorder characterized by the infiltration of inflammatory cells, including monocytes and macrophages, into the synovial joints, destroying cartilage and bone^1^. Peptidylarginine deiminase (PAD) enzymes catalyze calcium-dependent citrullination, which is a post-translational modification of arginine to citrulline^2^. Among the five PAD isoforms (PADs 1–4 and 6), PAD2 and PAD4 are the most intimately involved in the pathogenesis of RA^2–6^. Peptidylarginine deiminases can generate citrullinated proteins such as citrullinated forms of vimentin, fibrinogen, and α-enolase that play autoantigenic roles and lead to the production of anti-citrullinated protein antibodies (ACPAs), via the immune response in RA^7–12^. Therefore, PADs are implicated in the pathogenesis of seropositive RA.

On the other hand, single nucleotide polymorphisms (SNP) in the *PADI4* gene are independently associated with RA in European and Asian populations^5,13–16^. Furthermore, the *PADI4* risk allele contributes to the development of RA, regardless of anti-cyclic citrullinated peptide (anti-CCP) antibodies or erosive joint status^17^. In other words, the *PADI4* risk allele is involved in disease development, even in seronegative RA.

Peptidylarginine deiminases, including PAD4, can also self-deiminate via autocitrullination^18–20^. Although the immune response to autocitrullinated PADs as autoantigens also leads to the production of ACPAs^21^, the ACPA-independent functional role of autocitrullination in the pathogenesis of RA remains to be clarified.

We previously showed that epithelial-derived neutrophil-activating peptide 78 (ENA-78; CXCL5), a neutrophil recruiter, acquires monocyte-migratory ability after citrullination^22^. Therefore, we investigated the chemotactic function of recombinant human PAD4 (rhPAD4) with citrullination in vitro and in vivo to identify the ACPA-independent role of citrullinated PAD4 in the pathogenesis of RA. We also used a new, enzyme-linked immunosorbent assay (ELISA) to determine whether synovial fluid (SF) from patients with RA contains citrullinated PAD4 and examined relationships between levels of citrullinated PAD4 and anti-CCP antibodies.

## Methods

### Patients and healthy volunteers

Synovial fluid specimens obtained from the knee joints of patients with RA (n = 14) and osteoarthritis (OA) (n = 10) at The Jikei University Hospital were stored at − 80 °C. Blood samples for chemotaxis assays were collected from healthy volunteers (n = 5). The ethics committee at The Jikei University School of Medicine approved the study protocol [approval number 26-238 (7743)], and all patients and healthy volunteers provided written informed consent before enrollment in this study according to the principles of the Declaration of Helsinki.

### Autocitrullination of rhPAD4 in vitro

Recombinant human PAD4 (500 nM; Cayman Chemical, Ann Arbor, MI, USA) was incubated in 40 mM Tris–HCl with 2 mM CaCl_2_ (pH 7.4; reaction buffer) for 2 h at 37 °C. Autocitrullination was stopped using 0.5 M ethylenediaminetetraacetic acid (EDTA). Citrullinated rhPAD4 was stored at − 80 °C. For the chemotaxis assay and in vivo experiments outlined below, appropriate concentrations of citrullinated rhPAD4 was prepared by diluting a 500 nM solution.

### Western blotting citrullinated and rhPAD4

The final amounts of unmodified and citrullinated rhPAD4 proteins were adjusted to 200 ng/well. Unmodified and citrullinated rhPAD4 were resolved by 10% polyacrylamide gel electrophoresis, then blotted onto nitrocellulose membranes. Non-specific protein binding was blocked for 1 h at approximately 25 °C using 5% non-fat dried milk in Tris-buffered saline containing 0.1% Tween-20. Unmodified rhPAD4 was detected using a primary mouse anti-PAD4 monoclonal antibody (Merck KGaA, Darmstadt, Germany) diluted 1:2000, and secondary horseradish peroxidase (HRP)-linked anti-mouse IgG (Cell Signaling Technology, Danvers, MA, USA), diluted 1:1000. Citrullinated rhPAD4 on blots was chemically modified overnight at 37 °C to enable the detection of citrulline residues using human anti-modified citrulline antibody (Merck) as described by the manufacturer^22^, and then probed with 1:1000-diluted human anti-modified citrulline antibody for 1 h at approximately 25 °C. Bound antibodies were detected using 1:10,000-diluted HRP-conjugated goat anti-human IgG (Merck), then chemiluminescence was measured.

### Chemotaxis assays of monocytes and polymorphonuclear neutrophils (PMNs)

The chemotaxis of monocytes and PMNs isolated from the peripheral blood of healthy volunteers (n = 5), was analyzed using 48-well modified Boyden chambers (Neuro Probe Inc., Gaithersburg, MD, USA) as described^23,24^. The effects of unmodified rhPAD4, citrullinated rhPAD4, and reaction buffer (used for autocitrullination in vitro) on chemotaxis were determined. The respective negative and positive controls were phosphate-buffered saline (PBS) and 100-nM N-formyl-methionine-leucine-phenylalanine (fMLP; Sigma-Aldrich, St. Louis, MO, USA). Citrullinated rhPAD4 was prepared by incubating 500 nM unmodified rhPAD4 in the reaction buffer; the reaction was then stopped by adding 0.5 M EDTA. Unmodified rhPAD4 was prepared by first adding EDTA to the reaction buffer to chelate calcium and prevent the progression of autocitrullination, followed by adding the unmodified rhPAD4 to the reaction buffer. The reaction buffer used in the chemotaxis assay was prepared by mixing 40 mM Tris–HCl with 2 mM CaCl_2_ and 0.5 M EDTA. Unmodified or citrullinated rhPAD4 (0.1, 1, 10, 50, and 100 nM) were added as stimulants to the bottom wells of the chambers. The reaction buffer was diluted with PBS in the same way as the stimulants. Polyvinylpyrrolidone-free polycarbonate filters (Poretics Corp., Livermore, CA, USA) with 5- and 3-µm pores were placed in the assemblies for monocytes and PMNs, respectively, and 40 µL of cells (monocytes, 2.5 × 10^6^/mL; PMNs, 1.0 × 10^6^ cells/mL) in PBS with calcium and magnesium were placed in the top wells. Monocyte and PMN chemotaxis chambers were incubated in a 5% CO_2_ atmosphere at 37 °C for 90 and 35 min, respectively. The filters were fixed and stained with Diff-Quik (Baxter Healthcare Corp, Deerfield, IL, USA). Migrated cells were counted in 3 high-power fields (400×) per well in quadruplicate. The results are expressed as fold increase, obtained by dividing the number of cells that migrated to the stimuli by the number of cells that migrated to the negative control.

### Experimental murine model of inflammatory arthritis

The Institutional Animal Care and Use Committee of The Jikei University School of Medicine approved the experiments involving mice (protocol number 26-032). The animal experiments were performed in accordance with the relevant guidelines, especially the Animal Research: Reporting of In Vivo Experiments (ARRIVE) guidelines. Eight-week-old C57BL/6J female mice (Charles River Laboratories Japan, Inc., Kanagawa, Japan) were randomly assigned to three groups (n = 10/group) and housed in groups of five. They were maintained under a standard 12:12 h light/dark cycle with food and water available ad libitum. The sample size was selected based on our previous study^22^. The mice were randomly assigned at five mice per gauge by keepers who were blinded to the experimental details. Anesthetized mice received intra-articular injections in both knee joints, of which 20 μL included one of the following: reaction buffer containing 0.5 M EDTA, 500 nM unmodified rhPAD4, or 500 nM citrullinated rhPAD4. Unmodified rhPAD4 administrated to mice in the PAD4 group was prepared by first adding EDTA to the reaction buffer to prevent the progression of autocitrullination, followed by the addition of PAD4. All three solutions administered to the three groups contained 0.5 M EDTA and had an identical composition. The knee circumference (n = 20 joints/group) of mice that were randomly selected by K.Y. was blindly measured by H.I. before, and at 24 h after the intra-articular injection as described^22^, the mice were transcardially perfused with ice-cold PBS followed by PBS containing 4% paraformaldehyde under isoflurane anesthesia. The knee joints perfusion-fixed in PBS containing 4% paraformaldehyde were removed, decalcified in 10% EDTA (pH 7.0) for 5 days, and embedded in paraffin. Paraffin-embedded sections (3–4 μm) were stained with hematoxylin and eosin (HE), and immunohistochemically stained to detect monocytes/macrophages and PMNs with rat anti-mouse F4/80 antibodies (Bio-Rad Laboratories Inc. Hercules, CA, USA) diluted 1:500 and rat anti-mouse Ly6G/Gr1 antibodies (LSBio, Seattle, WA, USA) diluted 1:200, respectively. To detect M1 monocytes/macrophages that have infiltrated mouse synovial tissues, paraffin-embedded serial sections were immunohistochemically stained with rat anti-mouse F4/80 antibodies as a macrophage marker (BMA BIOMEDICALS, Basel, Switzerland) diluted 1:250 and rabbit anti-iNOS antibody as an M1 macrophage marker (GeneTex, CA, USA) diluted 1:250. The numbers of Ly6G-positive cells with segmented nuclei and F4/80-positive cells were counted in the top three high-power fields (400×) in order of the number of inflammatory cells (n = 10–20 knee joints/group). Data were collected in a blinded manner. The experiment was repeated twice, with similar results.

### Sandwich ELISA for PAD4 and citrullinated PAD4 in SF from patients with OA or RA

Concentrations of PAD4 in OA and RA SF were measured using human PAD4 ELISA Kits (Cayman Chemical), as described by the manufacturer. An ELISA for citrullinated PAD4 was designed to determine the absorbance of citrullinated PAD4 in OA and RA SF. Ninety-six-well plates (Corning Inc., Corning, NY, USA) were coated with mouse anti-human PADI4 capture monoclonal antibody (OriGene, Rockville, MD, USA), and incubated overnight at 4 °C. The plates were washed between the following steps with PBS containing 0.05% Tween-20 (wash buffer). Non-specific binding in the wells was blocked for 1 h using 1% Block Ace (KAC Co., Ltd., Kyoto, Japan), in MilliQ water. Synovial fluid diluted 1:1 with 0.4% Block Ace was added to the wells, then the plates were incubated for 2 h at approximately 25 °C, followed by rabbit anti-conjugated citrulline antibody (Bio-Rad Laboratories Inc.), diluted 1:1000 in 0.4% Block Ace for 2 h at approximately 25 °C and goat anti-rabbit IgG-(H + L) HRP conjugate secondary antibody (Proteintech Group Inc. Rosemont, IL, USA), diluted 1:1000 in 0.4% Block Ace for 2 h at approximately 25 °C. Tetramethylbenzidine chromogen was added to the wells, and the reaction was stopped with 2N H_2_SO_4_. Absorbance in the wells was measured at 450 nm using a plate reader, and the absorbance of the blank was subtracted from that of the samples.

### Statistical analysis

Differences in values between two groups were analyzed using Mann–Whitney *U* tests and among three or more groups using Kruskal–Wallis and Dunn multiple comparison post hoc tests. Correlations between values were assessed using Spearman Rank correlation coefficients. All data were statistically analyzed using GraphPad Prism version 4.0 (GraphPad Software Inc., San Diego, CA, USA). The results are shown as means ± standard deviation (SD). Values with P < 0.05 were considered statistically significant.

### Ethics approval and consent to participate

This study was approved by the ethics committee at The Jikei University School of Medicine [approval number: 26-238 (7743)]. The protocol for animal experiments was reviewed and approved by the Institutional Animal Care and Use Committee of the Jikei University [approval number: 26-032] and conformed to the Guidelines for the Proper Conduct of Animal Experiments of the Science Council of Japan (2006).

## Results

### Autocitrullination of rhPAD4 in vitro

**Figure 1:**
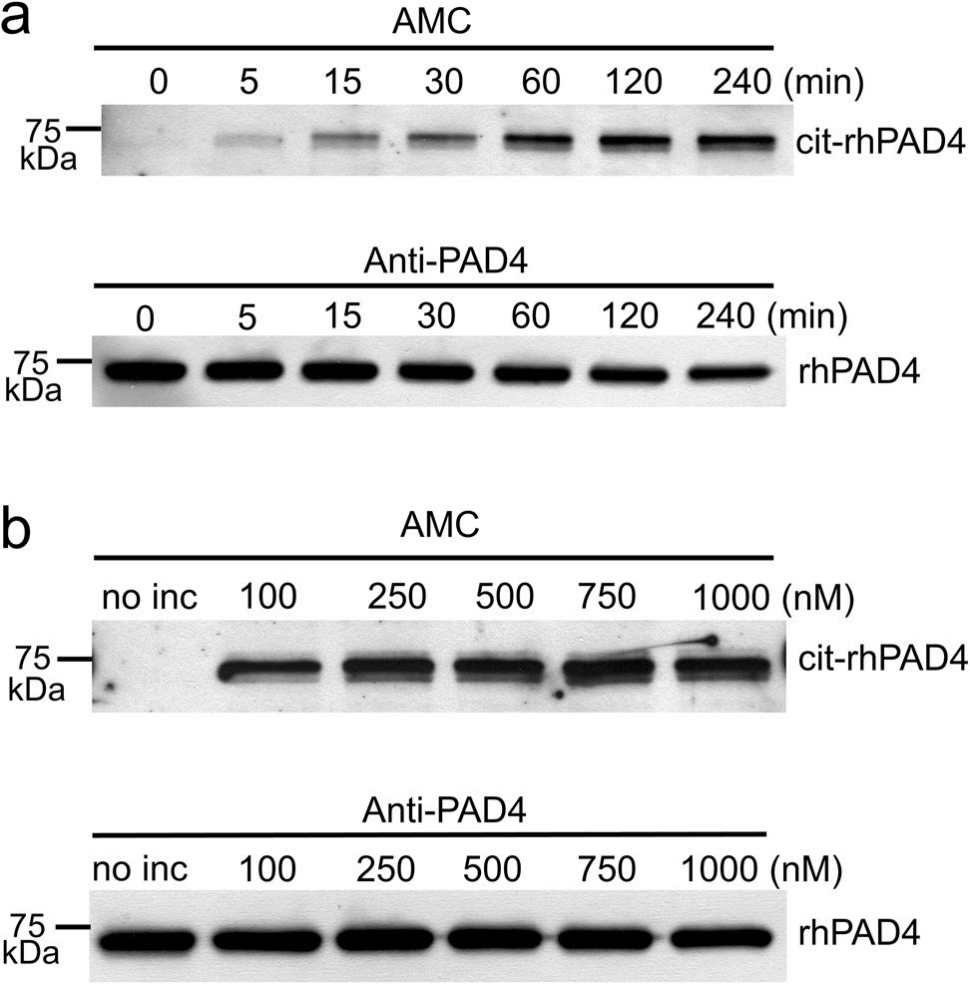
Autocitrullination of rhPAD4 in vitro. (**a**) Recombinant human PAD4 incubated in reaction buffer for 0–240 min. Citrullination of rhPAD4 (200 ng/lane) was confirmed by immunoblotting using anti-modified citrulline antibody (AMC). Recombinant human PAD4 (200 ng/lane) was detected by immunoblotting using anti-PAD4 antibody. (**b**) Recombinant human PAD4 (100–1000 nM) incubated in reaction buffer. Far left lane, rhPAD4 without incubation in reaction buffer. Citrullinated and unmodified rhPAD4 (both 200 ng/lane) were detected using AMC and anti-PAD4 antibodies, respectively. cit-rhPAD4: citrullinated recombinant human PAD4.

Western blotting confirmed that rhPAD4 autocitrullination started within 5 min, time-dependently increased, then peaked at 120 min (Fig. 1a). Autocitrullination of rhPAD4 was detected at all concentrations from 100 to 1000 nM but was more efficient at concentrations > 100 nM (Fig. 1b), but a white precipitate appeared at 1000 nM at 2 h after incubation. Therefore, citrullinated rhPAD4 used in all experiments was prepared at 500 nM and incubated for 120 min. A wide-range immunoblot version of Fig. 1 is presented in Supplementary Fig. S1.

### Citrullinated rhPAD4 recruited monocytes in vitro

**Figure 2:**
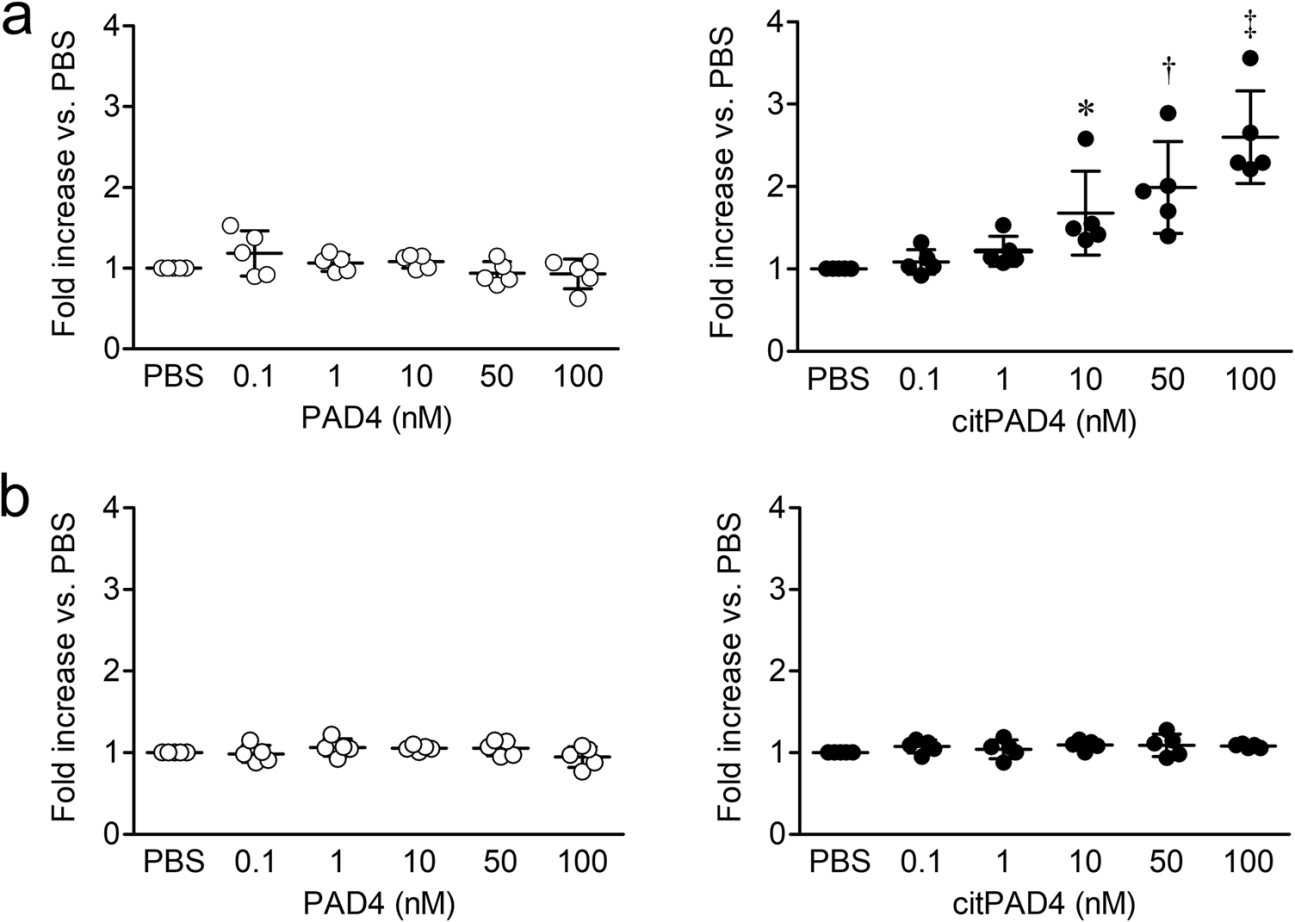
Chemotactic effects of the citrullinated form of rhPAD4 on monocytes in vitro. (**a**) Monocyte chemotaxis (n = 5). Monocyte migration increased in response to unmodified and citrullinated rhPAD4 (0.1, 1, 10, 50, and 100 nM) compared with negative control (phosphate-buffered saline [PBS]). Results are expressed as fold increase compared with negative control (PBS). The fold increase in response to fMLP as positive control was 4.92 ± 1.02. compared with PBS. (**b**) PMN chemotaxis (n = 5). PMN migration increased in response to unmodified and citrullinated rhPAD4 (0.1, 1, 10, 50, and 100 nM) compared with negative control (PBS). The fold increase in response to fMLP as positive control was 3.2 ± 1.04 compared with PBS. Values are shown as means ± SD. *P < 0.05, ^†^P < 0.01, and ^‡^P < 0.001.

The results of monocyte chemotaxis assays revealed a significantly higher dose-dependent increase in response to citrullinated rhPAD4, than to PBS at 10 (mean ± SD 1.68 ± 0.51; P < 0.05), 50 (1.99 ± 0.56; P < 0.01), and 100 (2.60 ± 0.56; P < 0.001) nM (Fig. 2a). However, the increases in responses to unmodified rhPAD4 and PBS did not significantly differ (Fig. 2a). The increase in response to fMLP as positive control was 4.92 ± 1.02. The increases in responses to reaction buffer (40 mM Tris–HCl and 2 mM CaCl_2_) used for rhPAD4 autocitrullination in vitro corresponding to each concentration of citrullinated rhPAD4 (0.1, 1, 10, 50, 100 ng/mL) were 0.97 ± 0.12, 0.99 ± 0.11, 1.01 ± 0.03, 1.01 ± 0.09, and 1.05 ± 0.07, respectively. The increases in responses to PBS and reaction buffer also did not significantly differ. The chemotactic responses of PMNs to unmodified and citrullinated rhPAD4 compared with PBS did not significantly differ (Fig. 2b). Compared with PBS, the increase in response to fMLP as positive control was 3.2 ± 1.04.

### Citrullinated rhPAD4 amplified the inflammatory response in mouse joints by monocyte migration

**Figure 3:**
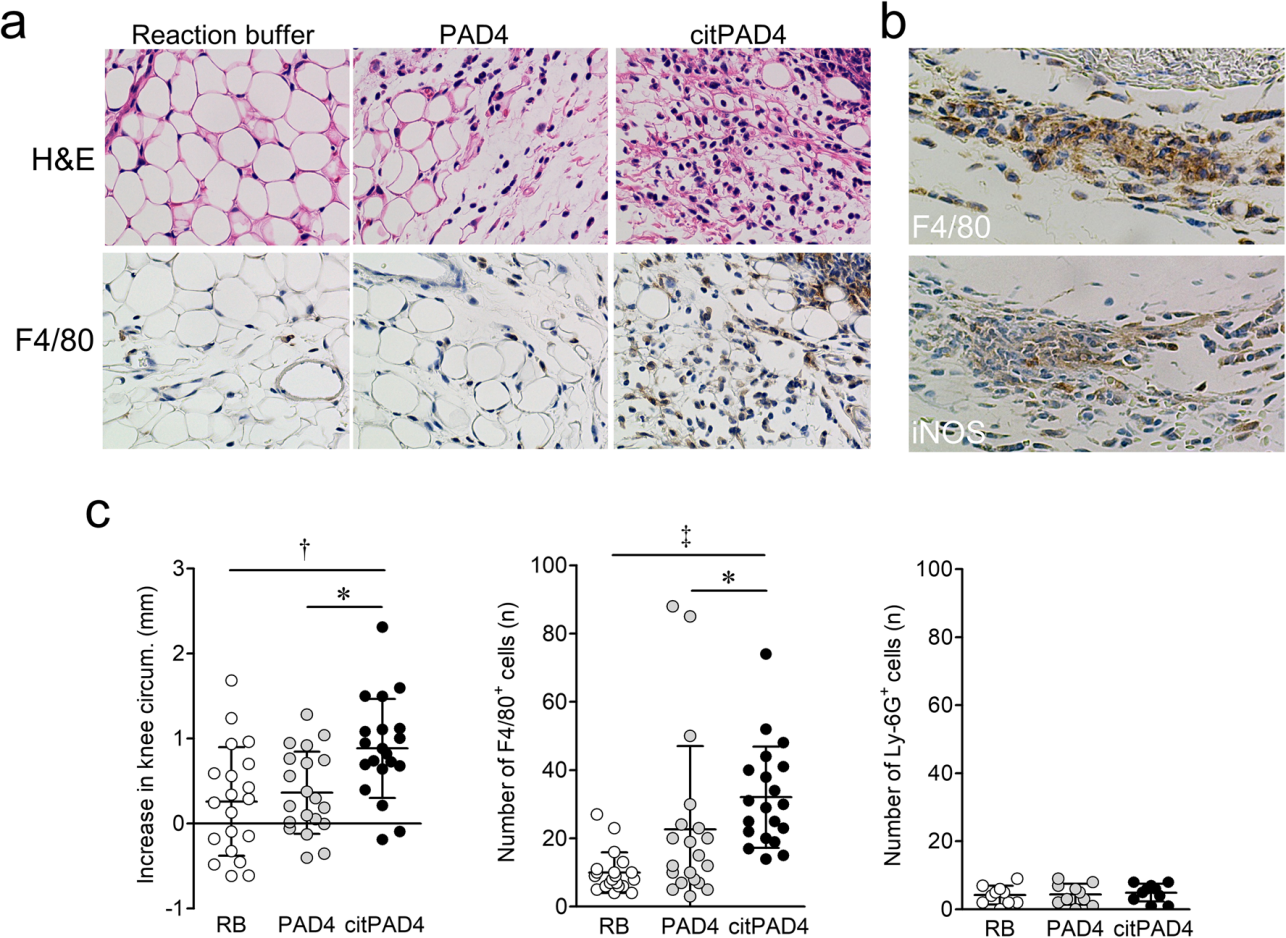
The effects of citrullinated rhPAD4 on C57BL/6J mice via monocyte migration in inflammatory arthritis. (**a**) Hematoxylin & eosin (H&E) and immunohistochemical staining for F4/80 show inflammatory and F4/80-positive cells infiltrating synovial tissues of mice given intra-articular injections of reaction buffer (RB), unmodified, or citrullinated rhPAD4 (Original magnification × 400). (**b**) Immunohistochemical staining show monocyte/macrophage marker F4/80-positive cells (upper panel) and M1 macrophage marker iNOS-positive cells (lower panel) infiltrating synovial tissues of mice given intra-articular injections of citrullinated rhPAD4 (Original magnification ×400). (**c**) Increased knee circumference between days 0 and 1 (n = 20 knee joints/group), numbers of F4/80 (n = 20 knee joints/group), and Ly6G-positive cells with segmented nuclei infiltrating synovial tissues (n = 10 knee joints/group) of mice with intra-articular injections of RB, unmodified, or citrullinated rhPAD4. Values are shown as means ± SD. *P < 0.05, ^†^P < 0.01, and ^‡^P < 0.001.

The knee circumference significantly increased in the groups given citrullinated rhPAD4 (mean ± SD 0.885 ± 0.582 mm), compared with reaction buffer (0.260 ± 0.641 mm; P < 0.01) and unmodified rhPAD4 (0.363 ± 0.108 mm; P < 0.05) in vivo (Fig. 3c). We then examined whether citrullinated rhPAD4 induces monocyte migration in a mouse model. Sections of mouse synovial tissues stained with HE revealed massive infiltration of mononuclear cells in the citrullinated rhPAD4, compared with the reaction buffer and unmodified rhPAD4 groups (Fig. 3a). Significantly more F4/80-positive cells infiltrated knee synovial tissues (n = 20 knee joints/group) in the group given citrullinated rhPAD4 (mean ± SD 32.1 ± 14.9) than in those given the reaction buffer (10.0 ± 6.0; P < 0.001) or unmodified rhPAD4 (22.7 ± 24.4; P < 0.05; Fig. 3a, c). Moreover, F4/80-positive cells that migrated to mouse joints in the citrullinated rhPAD4 group also included the M1 macrophage marker iNOS (Fig. 3b). In contrast, the numbers of infiltrative Ly6G-positive cells with segmented nuclei in knee synovial tissues (n = 10 knee joints/group) did not significantly differ among the three groups (Fig. 3c).

### Levels of PAD4 and citrullinated PAD4 were higher in the SF in RA compared to that in OA

**Figure 4:**
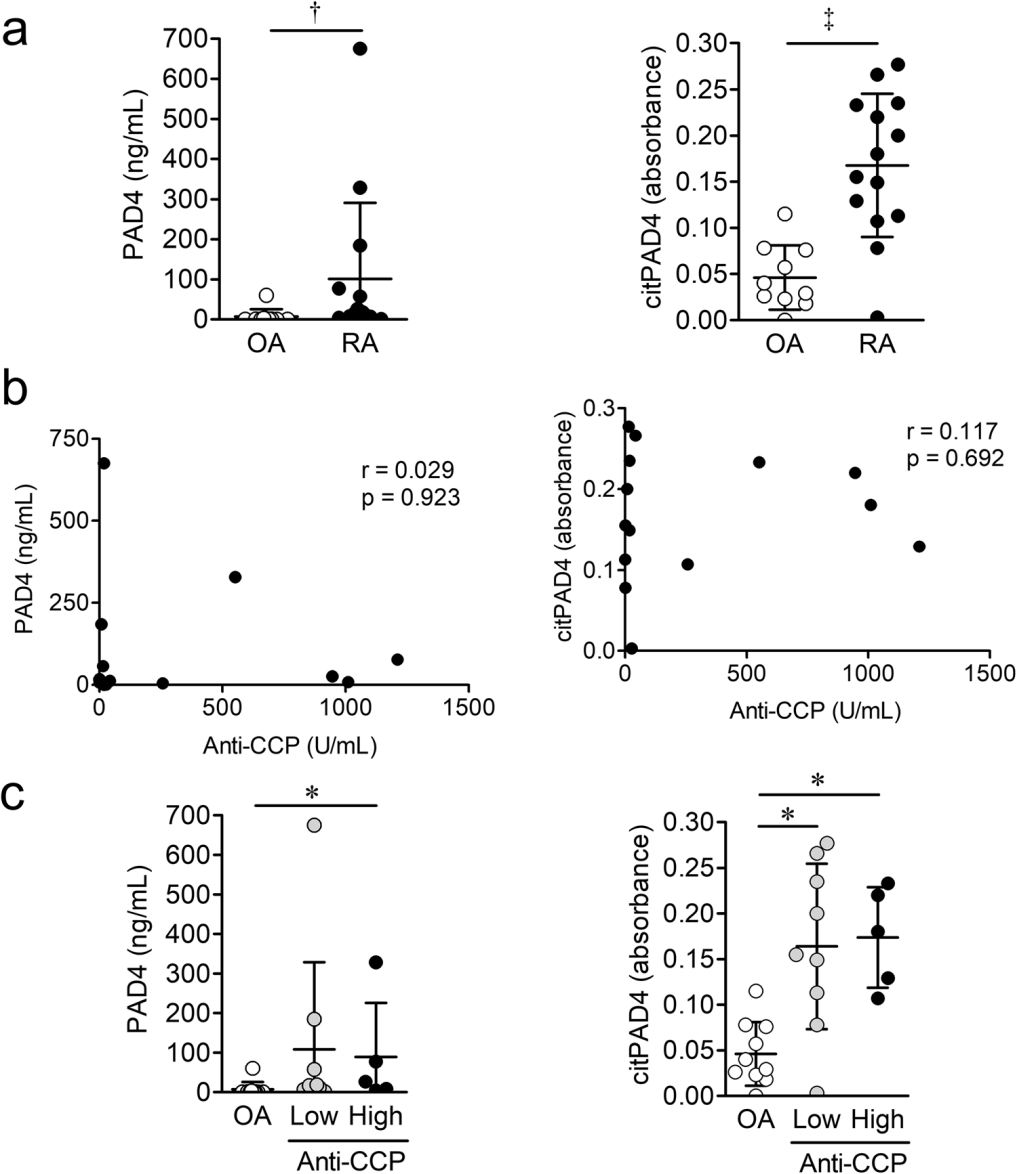
Levels of PAD4 and citrullinated PAD4 (citPAD4) protein in synovial fluid (SF) from patients with osteoarthritis (OA) or rheumatoid arthritis (RA), and relationship with anti-CCP titers. (**a**) Concentrations of PAD4 and absorbance of citPAD4 in SF from patients with OA (n = 10) or RA (n = 14). (**b**) Correlations between anti-cyclic citrullinated peptide (CCP) levels and PAD4 concentrations or absorbance of citPAD4 in SF from patients with RA. (**c**) Concentrations of PAD4 and absorbance of citPAD4 in SF from patients with RA with low (0 ≤ 100 U/mL, n = 9) and high (> 100 U/mL, n = 5) titers of anti-CCP. Values are shown as means ± SD. *P < 0.05, ^†^P < 0.01, and ^‡^P < 0.001.

The concentrations of PAD4 in SF from patients with OA or RA were measured using commercial ELISA kits (Cayman Chemical). The concentration of PAD4 was significantly higher in SF from patients with RA than OA (mean ± SD 101.2 ± 189.2 and 7.3 ± 18.6 ng/mL, respectively; P = 0.0077) (Fig. 4a). The absorbance of citrullinated PAD4 in SF from patients with OA or RA was measured using our newly developed ELISA. The absorbance of citrullinated PAD4 was significantly higher in SF from patients with RA than OA (0.168 ± 0.078 and 0.046 ± 0.035, respectively; P = 0.0009; Fig. 4a).

### Relationship between serum anti-CCP and PAD4 or citrullinated PAD4 protein levels in SF from patients with RA

Serum levels of anti-CCP did not correlate with either PAD4 or citrullinated PAD4 in SF (Fig. 4b). The patients with RA were divided according to whether they had low (≤ 100 U/mL; n = 9) or high (> 100 U/mL; n = 5) anti-CCP titers^25^. The level of PAD4 was significantly greater in SF from the patients with RA and a high anti-CCP titer than in SF from patients with OA (means ± SD; 88.9 ± 137.0 and 7.3 ± 18.6 ng/mL, respectively; P < 0.05; Fig. 4c). The levels of SF-citrullinated PAD4 in the low-titer group, including anti-CCP negative patients with RA (n = 3), and in the high-titer group were significantly higher than those in patients with OA (0.164 ± 0.091, 0.174 ± 0.055, and 0.046 ± 0.035 ng/mL, respectively; P < 0.05; Fig. 4c). The levels of SF-citrullinated PAD4 in the low and high titer groups did not significantly differ.

## Discussion

Autocitrullination of rhPAD4 increased in a time-dependent manner in vitro. However, compared to the band of unmodified rhPAD4 in Fig. 1a, it appears that the intensity of the band recognized by the anti-PAD4 antibody slightly weakens as citrullination proceeds, suggesting that some types of citrullinated forms of PAD4 are no longer recognized by the anti-PAD4 antibody.

We then investigated whether PAD4 autocitrullination recruits inflammatory cells, such as monocytes and PMNs. The findings of our chemotactic assays showed that autocitrullination conferred monocyte chemotactic properties upon the PAD4 enzyme. The pathogenic role of autoantibodies against citrullinated proteins is under further investigation. For instance, ACPAs induce bone loss in mice via interleukin-8-dependent and citrulline-specific mechanisms^26,27^, and accelerate synovial fibroblast migration^28^. However, the role of citrullinated proteins, other than as autoantigens, remains unclear. Citrullination provokes protein unfolding due to a decrease in the net positive charge, the loss of potential ionic bonds, and interference by H bonds. These result in the modification of conformational and functional features of the protein^29^. Citrullinated forms of fibronectin and vimentin promote the secretion of pro-inflammatory cytokines from fibroblast-like synoviocytes in patients with RA^30,31^. We previously showed that ENA-78/CXCL5, which stimulates neutrophil chemotaxis, acquires monocyte-migratory ability after citrullination by binding to CXCR2 and CXCR1^22^. Although we did not examine chemokine receptors bound to citrullinated rhPAD4, we found that the citrullinated form of rhPAD4 enzyme also acquired monocyte chemotactic properties. Further studies are required to clarify the mechanism of the monocyte-migratory response induced by citrullinated PAD4.

We also showed that citrullinated rhPAD4 amplified inflammation in mouse joints. The *PADI4* haplotype, which is associated with susceptibility to RA, augments the production of citrullinated peptides that act as autoantigens^3^. However, the *PADI4* allele is an independent genetic risk for radiographic progression in Japanese patients with RA and contributes to its development independently of anti-CCP antibodies^17,32^. We found that more F4/80-positive cells infiltrated the synovial tissues of mouse knee joints in the citrullinated group than in the unmodified rhPAD4 group. Among monocyte/macrophage marker F4/80-positive cells, M1 macrophage marker iNOS-positive cells, which are mainly involved in pro-inflammatory responses^33^, were detected in the synovial tissues of mice in the citrullinated PAD4 group. In contrast, the numbers of the few PMNs found in the synovial tissues of these groups did not significantly differ. We confirmed that the autocitrullination of rhPAD4 enhanced mouse-articular inflammation via the monocytes but not PMN infiltration of the synovial tissues, suggesting that autocitrullination contributes to RA pathogenesis by facilitating the recruitment of monocytes/macrophages, including M1 macrophages, which produce crucial inflammatory cytokines and promote a pro-inflammatory response.

We found higher concentrations of PAD4 in SF from joints with RA compared to those with OA. Foulquier et al.^34^ identified PAD4 by immunoblotting in synovial tissue samples from 13 of 16 patients with RA and in all four patients with other forms of arthritis, but in only two of seven with OA. Ishigami et al.^35^ found significantly elevated PAD4 (0–5.07 ng/mL) in plasma from patients with RA than from patients with OA and healthy individuals. On the other hand, the mean concentration of PAD4 in SF (0.6–675.4 ng/mL) in the present study was ~ 100-fold higher than that in plasma from patients with RA in the study by Ishigami.

To the best of our knowledge, this is the first study to quantify citrullinated PAD4 in human biological fluids. Levels of citrullinated PAD4 were significantly higher in SF from RA than OA, regardless of anti-CCP titers, even in patients with no or low titers of anti-CCP. T cells, B cells, macrophages, neutrophils, fibroblast-like and endothelial cells in synovial tissues from patients with RA all express PAD4^36^. Neutrophil extracellular traps released from nuclear material result in the extracellular release of PAD4 in patients with RA^37^. Abundant neutrophils maintain the constant release of active PAD, including PAD4, for extracellular citrullination in the articular cavity in RA^38^, suggesting that this is a critical site for persistent autocitrullination. Therefore, PAD4 autocitrullination might be involved in the pathogenesis of not only seropositive but also seronegative RA.

The limitations of the current study include the lack of a detailed investigation into ELISA standards, spike and recovery tests, and the establishment of a cut-off value for the in-house ELISA system used to detect citrullinated forms of PAD4. It is unclear whether all citrullinated forms of PAD4 were detected using this ELISA system, and further verification is required.

**Figure 5:**
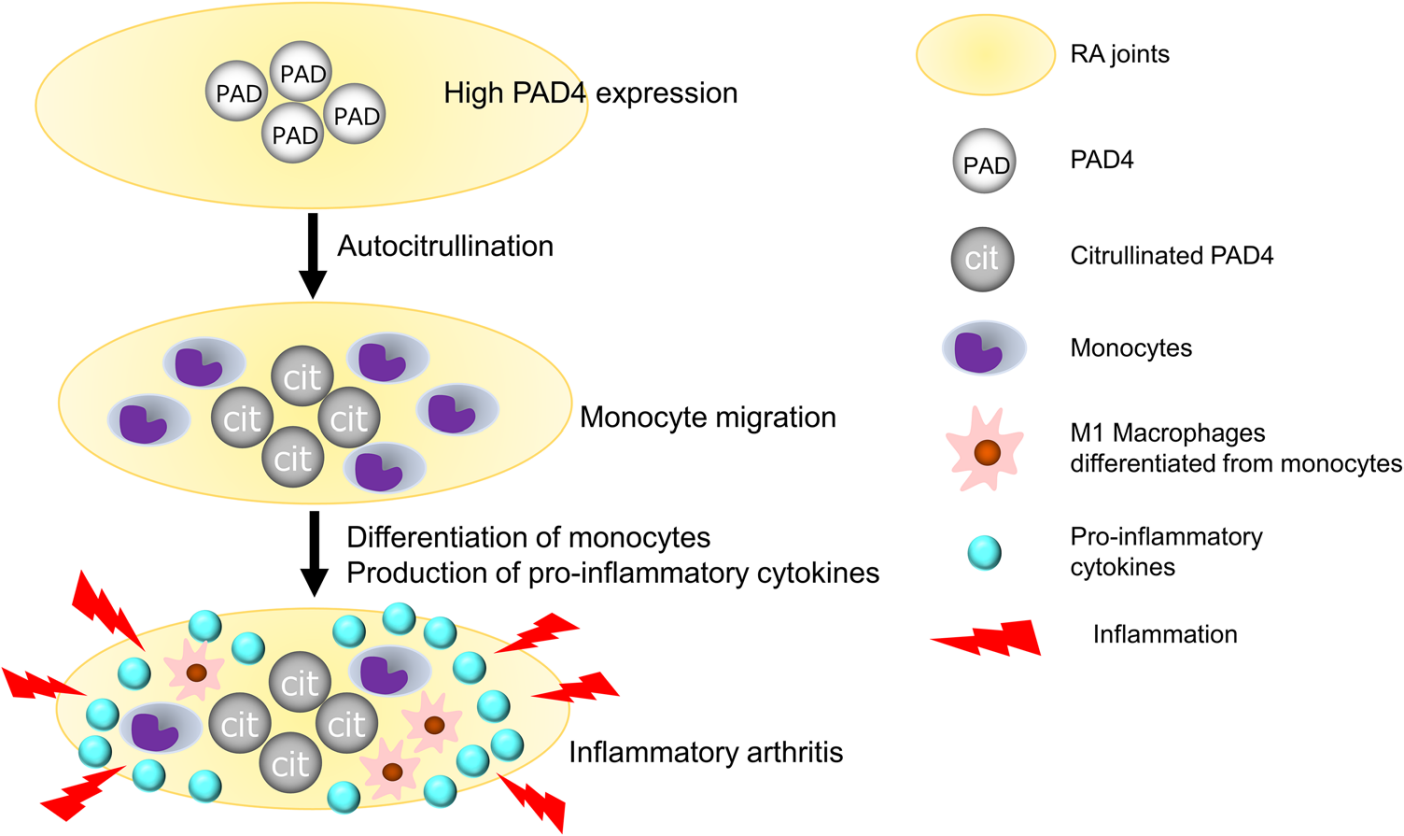
A model illustrating the ACPA-independent mechanism in the pathogenesis of RA.

In conclusion, autocitrullination conferred monocyte-chemotactic activity on rhPAD4, which amplified articular inflammation in a mouse model through monocyte recruitment. Levels of citrullinated PAD4 were significantly elevated in SF from patients with RA compared with OA, independently of the anti-CCP titer. The chemokine-like effect of citrullinated PAD4 is potentially relevant to the ACPA-independent mechanism of RA pathogenesis (Fig. 5).

## Supplementary Information

Supplementary Figure S1.

## Data Availability

All data included in the present study are available from the corresponding author on reasonable request.
