# Supplementary material for: Autocitrullination confers monocyte chemotactic properties to peptidylarginine deiminase 4
Source: Sci Rep. 2023 May 9;13:7528. doi: 10.1038/s41598-023-34469-1 (PMC10169855; doi:10.1038/s41598-023-34469-1)
Supplement: Supplementary file 1 — Supplementary Figure S1. [file 41598_2023_34469_MOESM1_ESM.docx]

**Autocitrullination** **confers monocyte chemotactic properties to peptidylarginine deiminase 4**

Ken Yoshida^1,^*, Haruyasu Ito^1^, Daisaburo Kurosaka^2^, Ryo Ikeda^2^, Kentaro Noda^1^, Mitsuru Saito^2^ & Daitaro Kurosaka^1^


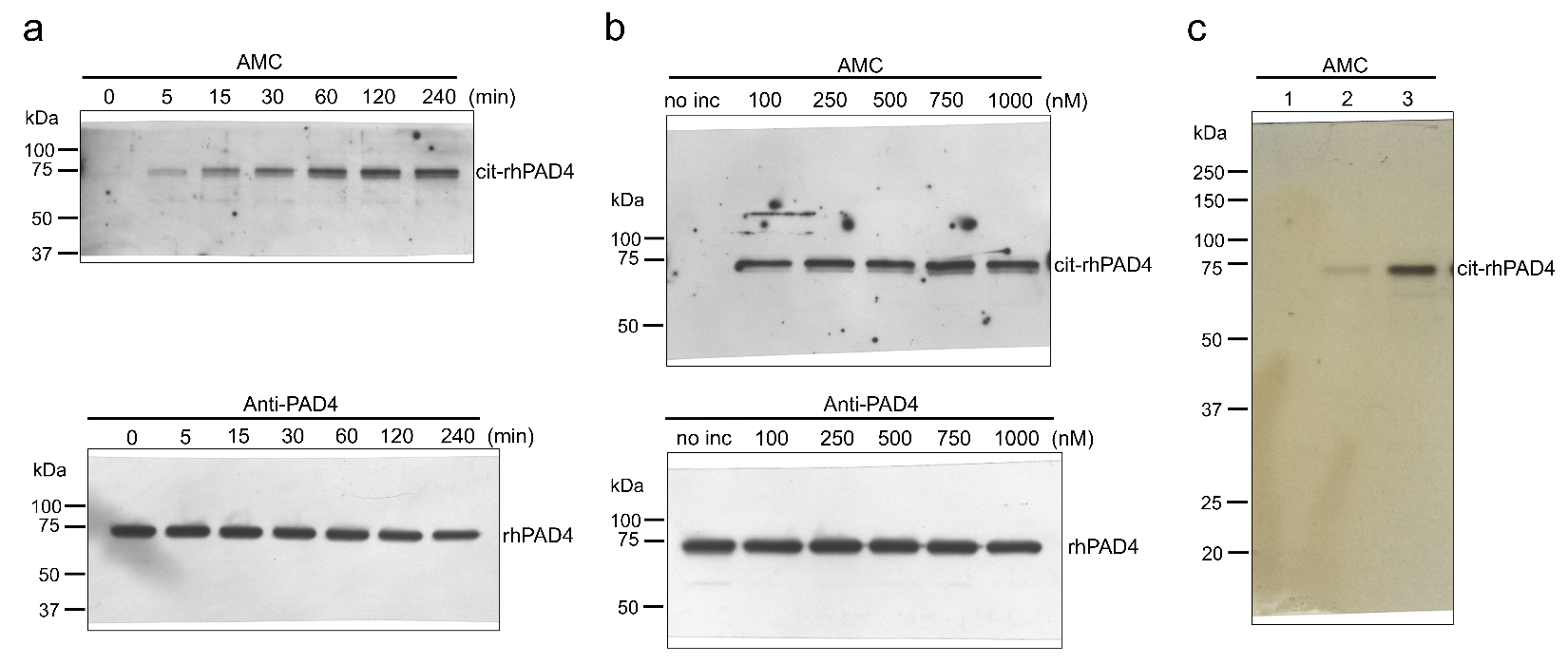


**Supplementary Figure S1.** An uncropped immunoblot version of Figure 1.

Supplementary Figures S1a and S1b show the uncropped immunoblots shown in Figure 1. The upper and lower portions of the original film, where these recombinant proteins are not detected, were cut off, but these uncropped blots demonstrate an absence of cross-reactive bands above and below the 75 kDa range. A commercially available, purified recombinant human PAD4 (rhPAD4) was used in these experiments. Unmodified rhPAD4 is autocitrullinated; thus, the recombinant protein bands detected by immunoblotting were in the range of 75 kDa. The recombinant proteins were blotted onto nitrocellulose membranes and detected via exposure to chemiluminescence films. In addition, Supplementary Figure S1c displays a film covering a broader range of molecular weights. This film was developed during our preliminary immunoblotting experiments. Lanes 1, 2, and 3 show unmodified rhPAD4 (200 ng/lane), citrullinated rhPAD4 (100 ng/lane), and citrullinated rhPAD4 (200 ng/lane), respectively. AMC: anti-modified citrulline antibody; cit-rhPAD4: citrullinated recombinant human PAD4; no inc: no incubation in reaction buffer (unmodified rhPAD4)
